# Glycocalyx at the host–virus interface: a double-edged sword in virus infection and tissue damage

**DOI:** 10.3389/fmolb.2026.1876119

**Published:** 2026-07-10

**Authors:** Vaibhav Tiwari, Anjaly Kappen, Alex Paul, James Elste, Chunyu Wang, Michelle Swanson-Mungerson, Michael V. Volin, Fuming Zhang

**Affiliations:** 1 Department of Microbiology and Immunology, College of Graduate Studies, Midwestern University, Downers Grove, IL, United States; 2 Departments of Chemical and Biological Engineering, Center for Biotechnology and Interdisciplinary Studies, Rensselaer Polytechnic Institute, Troy, NY, United States

**Keywords:** antiviral, glycocalyx, glycocalyx regeneration, heparan sulfate, inflammation, viral entry, virus host cell interactions

## Abstract

Viral entry is traditionally viewed as a receptor-mediated event, yet this paradigm overlooks the glycocalyx (GLX) - a dynamic, multifunctional layer of glycans, proteoglycans, and glycolipids that forms the host’s first molecular interface with pathogens. Emerging evidence positions the GLX as a bidirectional regulator of infection, capable of both restricting viral access and orchestrating virion capture, receptor clustering, and entry pathway selection. In herpes simplex virus (HSV) infection, cell-type specific GLX architectures on epithelial, endothelial, and immune cells dictate viral tropism, migration, antigen sensing, and immune synapse formation. Infection and inflammation remodel or shed the GLX, perturbing receptor organization, signaling networks, and immune function, thereby contributing to tissue pathology. Beyond its barrier role, the GLX acts as a mechanochemical sensor, integrating environmental cues to coordinate tissue homeostasis and repair. Here, we synthesize recent advances elucidating how GLX composition, spatial organization, and mechanical properties shape viral entry and host responses. We further highlight emerging biomimetic and synthetic strategies to manipulate the GLX, offering new avenues to interrogate virus–host interactions and therapeutic intervention. Collectively, these perspectives redefine the GLX as a central regulator of herpesvirus pathogenesis, functioning simultaneously as a protective shield and facilitator of infection.

## 1 Introduction

Viral entry represents the decisive first step in infection, establishing the spatial and temporal context in which all subsequent stages of the viral life cycle unfold (Spear et al., 1992; Valerio et al., 2025; Dimitrov, 2004; Mercer et al., 2010; White, 1992). For decades, the prevailing framework for understanding this process has been dominated by receptor-centric models, where viral surface proteins bind specific host cell receptors in a “lock-and-key” manner to initiate membrane fusion or endocytosis (Grove and Marsh, 2011; Speranza, 2026). While this paradigm has been instrumental in characterizing critical receptor domains (Terry-Allison et al., 1998; Yoon et al., 2003; Spear et al., 2006; Manoj et al., 2004; Warner et al., 1998), mapping tissue-specific tropism (Connolly et al., 2021; McClain and Fuller, 1994; Norris et al., 2025) and developing receptor-targeted antivirals (Majdoul and Compton, 2022; Fan et al., 2025; Hilterbrand et al., 2021; Ulrich et al., 2024; Elste et al., 2025; Koelle and Corey, 2003), this simplified view fails to capture the true complexity of the host cell surface. Increasingly, it appears insufficient to explain the profound variability in viral infectivity across different physiological states, tissues, and disease conditions. All mammalian cells are enveloped by a glycocalyx (GLX) - a dense, hydrated, and highly heterogeneous carbohydrate-rich layer composed of glycoproteins, proteoglycans (PGs), glycolipids, and polymeric mucins (Mockl, 2020; Domma et al., 2024; Ziarnik et al., 2025). Far from static molecular decoration, the GLX is now recognized as a regulatory organelle that actively shapes membrane architecture, immune regulation, and pathological outcomes (Moore et al., 2021; Dancy et al., 2024; Sun et al., 2023; Potje et al., 2021; Veraldi et al., 2022; Meneses et al., 2017). As a mesoscale structure extending hundreds of nanometers from the plasma membrane, the GLX far exceeds the size of most viral receptors and rivals the dimensions of the viral particles themselves (Ziarnik et al., 2025; Veraldi et al., 2022). Consequently, viruses approaching a host cell do not encounter receptors directly; instead, they must first navigate, deform, and interact with the GLX (Ziarnik et al., 2025). This realization necessitates a fundamental rethinking of viral entry as a biophysically regulated process, governed by polymer physics and electrostatics rather than purely biochemical recognition events.

Herpes simplex virus type 1 (HSV-1), a ubiquitous enveloped DNA virus with broad cellular tropism, serves as an incisive model for probing GLX-mediated viral entry (Whitley and Roizman, 2001). HSV-1 entry proceeds through a finely orchestrated, multistep cascade in which viral glycoproteins gB, gD, and gH/gL engage multiple distinct host receptors (Spear et al., 1992; Spear, 2004). Critically, the initial attachment is dominated by interactions with heparan sulfate (HS) glycosaminoglycans, which are central constituents of the GLX (Spear et al., 1992). This positions HSV-1 at the nexus of glycobiology and membrane mechanics, where the GLX dictates receptor nanoclustering, local membrane curvature, and spatial organization parameters that collectively modulate the threshold for productive viral fusion. Beyond steric regulation, the highly anionic HS generates long-range electrostatic landscapes that guide viral approach and orient envelope glycoproteins bearing complementary cationic patches, optimizing surface scanning and receptor engagement (Tiwari et al., 2015). Importantly, the GLX is dynamically remodeled by cellular metabolism, enzymatic activity, and environmental stressors such as hyperglycemia or chronic inflammation. Perturbation in GLX integrity disrupts both steric and electrostatic control, reshaping the local viral microenvironment and markedly enhancing susceptibility (Taghavi et al., 2022; Mir and Legradi, 2025; Masenga et al., 2024; Tiwari et al., 2004). This mechanistic framework illuminates why viral infection severity is often tightly linked to host comorbidities, a phenomenon not explained solely by receptor abundance, and underscores the GLX as a tunable determinant of viral tropism and entry efficiency.

In parallel, it is essential to recognize that the current understanding of GLX–virus interactions emerge from a heterogeneous methodological landscape, rather than a single experimental paradigm. Reductionist biochemical and classical cell line-based systems (including receptor binding assays, plaque reduction assays, and overexpression models in HeLa or Vero cells) provide high mechanistic resolution but inherently fail to reproduce the full physiological architecture of the GLX, including native glycosylation complexity, membrane tension, and polymer brush organization (Purcell and Godula, 2019; Chin-Hun Kuo et al., 2018; Shurer et al., 2019). Primary human epithelial and stromal cells partially restore this native context but remain limited by donor variability, restricted lifespan, and incomplete representation of tissue-scale microenvironmental diversity (Chin-Hun Kuo et al., 2018). More recently, three-dimensional organoids and organ-on-chip platforms have significantly improved physiological relevance by recapitulating tissue-specific GLX architecture, fluid shear stress, and multicellular signaling networks, although they still do not fully capture systemic immune and metabolic coupling present *in vivo* (Ingber, 2022). Synthetic GLX engineering approaches, including polymer brush systems and glycoengineered cell surfaces, enable precise and causal interrogation of GLX parameters such as chain length, grafting density, and surface charge; however, these systems necessarily simplify native compositional heterogeneity (Kuo and Paszek, 2021). Complementarily, *in silico* frameworks including molecular dynamics simulations, polymer physics-based models, and electrostatic field mapping provide quantitative predictions of virus GLX interactions, yet remain dependent on parameter assumptions, force-field constraints, and simplified boundary conditions (Ziarnik et al., 2025). Collectively, these approaches form a multiscale investigative continuum, where each model captures distinct aspects of GLX biology, but none alone fully recapitulates the integrated *in vivo* environment. Accordingly, interpretation and extrapolation of findings must explicitly account for the inherent strengths and limitations of each system. Importantly, this recognition does not diminish the value of existing models; rather, it underscores that GLX biology and by extension viral entry and trafficking can only be accurately understood through convergence of complementary experimental and computational perspectives. Despite its fundamental role in cellular defense, the GLX has historically been underexplored in experimental virology, largely due to the inherent fragility and structural complexity of this extracellular layer. Recent advances, however, are rapidly transforming this landscape: super-resolution imaging, atomic force microscopy, and precise glycoengineering now enable high-resolution visualization and controlled manipulation of GLX components (Purcell and Godula, 2019; Kolesov et al., 2023; Wang et al., 2024a; Huang et al., 2018). In particular, chemical biology provides powerful tools to conceptualize viral entry as a tunable, quantitative system. By engineering cells with defined GLX architectures ranging from synthetic polymer brushes to overexpressed mucins, researchers can systematically dissect how physical barrier modulate fusion kinetics and infection outcomes (Liu et al., 2024; Delaveris et al., 2020; Honigfort et al., 2021; Kohout et al., 2022; Chighizola et al., 2022). Looking ahead, leveraging physiologically relevant primary human cell cultures and organoid-based models provides an unprecedented opportunity to identify the authentic molecular determinants of viral entry in human tissues, overcoming the limitations of conventional transformed cell line studies. In this review, we consolidate emerging evidence highlighting the GLX as a central regulator of viral entry, with HSV-1 serving as a paradigmatic model. We propose that viral entry is a multi-step, GLX-mediated process governed by an interplay of steric hindrance, electrostatics, and membrane mechanics, offering a predictive framework to guide the development of next-generation antivirals and tissue-specific therapeutic interventions.

## 2 Structural organization of the glycocalyx

### 2.1 Molecular components

The molecular architecture of the GLX constitutes a complex, hierarchical assembly where the spatial and biochemical interplay of diverse macromolecules dictates the functional topography of the cell surface. Rather than a static or uniform coating, the GLX is a structurally heterogeneous and dynamically regulated interface composed of diverse carbohydrate-bearing macromolecules (Mockl, 2020; Delaveris et al., 2020). These constituents vary significantly in their hydrodynamic radii, conformational flexibility, charge density, and membrane-anchoring mechanisms, collectively forming a multifunctional biophysical sieve that integrates biochemical signaling with steric barrier properties. Understanding this composition is critical, as the GLX serves as the primary metabolic and physical checkpoint for viral particles attempting to access the plasma membrane (Mockl, 2020; Moore et al., 2021; Dancy et al., 2024). At its structural core, the GLX consists of proteoglycans (PGs), glycosaminoglycan (GAG) chains, glycoproteins, glycolipids, and mucins, each contributing distinct structural and functional attributes (Mockl, 2020; Moore et al., 2021; Dancy et al., 2024). PGs serve as primary scaffolds, featuring core proteins covalently linked to GAG side chains predominantly heparan sulfate and chondroitin sulfate whose high degree of sulfation imparts a potent polyanionic character to the cell surface. This negative charge density facilitates the electrostatic sequestration of viral ligands and modulates the local ionic environment (Mockl, 2020; Dancy et al., 2024). Simultaneously, membrane-bound and secreted mucins contribute massive, heavily O-glycosylated scaffolds enriched in terminal sialylated and sulfated glycans that extend far into the extracellular space, creating a dense glycan brush that regulates viral access through both specific glycan–protein interactions and nonspecific steric exclusion (Moore et al., 2021; Delaveris et al., 2020; Corfield, 2015; Tailford et al., 2015). Because the relative abundance and turnover rates of these components vary widely between cell types, tissues, and physiological states, the resulting GLX architecture serves as a primary determinant of cell-specific viral susceptibility.

### 2.2 Proteoglycans (PGs) as structural scaffolds

Proteoglycans (PGs) function as the primary structural backbone of the glycocalyx (GLX), anchoring and organizing the complex carbohydrate network at the cell surface (Kaur and Harris, 2023; Reitsma et al., 2007). These macromolecules consist of a central core protein to which one or more glycosaminoglycan (GAG) chains are covalently attached (Jin et al., 2021; Mathis et al., 2021). The major GLX-associated GAGs include heparan sulfate, chondroitin sulfate, dermatan sulfate, keratan sulfate, and hyaluronic acid, which are displayed on the cell surface through proteoglycan core proteins or as free polysaccharides in the extracellular matrix (Lindahl et al., 2015). Among the principal cell-surface PGs, Syndecans and glypicans play central roles in organizing GLX architecture and regulating host–pathogen interactions. Syndecans are transmembrane PGs whose cytoplasmic domains connect directly to the actin cytoskeleton, enabling extracellular GLX organization to influence intracellular signaling, membrane dynamics, and mechanotransduction (Kaur and Harris, 2023; Mathis et al., 2021). These structural differences have important implications for receptor clustering, signal transduction, mechanosensing, and the cellular processes that govern viral attachment, uptake, and entry (Vinzant et al., 2025). From a virological perspective, PGs serve two key roles. First, they provide high-density anchoring sites for GAG chains, thereby defining the thickness and density of the GLX (Mockl, 2020). Second, they function as initial viral attachment platforms, particularly for viruses that bind HS (Shukla and Spear, 2001; Oh et al., 2010). In the case of HSV-1, initial attachment to heparan sulfate proteoglycans (HSPGs) serve to concentrate virions on the cell surface. This recruitment occurs without immediately triggering fusion, underscoring the scaffolding role of the GLX in regulating the early stages of entry. Remarkably, specific 3-O-sulfated (3-OS) moieties within the HS chain can function as a high-affinity receptor for HSV-1 glycoprotein D (gD). This interaction is potent enough to trigger virus–host cell fusion even in the absence of traditional protein receptors such as nectin-1 or HVEM (Spear, 2004). Despite these insights, a critical gap remains as we do not yet fully understand how the acute or chronic GLX remodeling characterized by shedding and enzymatic degradation affects the expression or accessibility of these 3-O-sulfated motifs. Aging and chronic conditions such as diabetes, obesity, and cardiovascular disease are known to induce significant GLX degradation and shedding (Chelazzi et al., 2015; Wang et al., 2024b), accompanied by altered expression of heparan sulfate sulfotransferases and enhanced activity of GLX-remodeling enzymes, including heparanase and extracellular sulfatases (SULF1 and SULF2) (Xu and Esko, 2014; Vlodavsky et al., 2016; Kim et al., 2021). Together, these changes remodel heparan sulfate density and sulfation patterns, potentially may influence viral attachment, receptor accessibility, and host susceptibility to infection. In addition, Flavivirus NS1, a secreted glycoprotein, drives tissue-specific vascular leakage by binding to endothelial cells and disrupting the endothelial GLX (Puerta-Guardo et al., 2019). This leads to increased permeability across multiple organs, reflecting virus-specific disease patterns (Puerta-Guardo et al., 2019). These findings highlight NS1-mediated GLX damage as a key mechanism of pathogenesis and a potential target for antiviral therapies. Interestingly, HSV-1 hijacks the GLX lectin galectin-3 to promote viral attachment and entry into corneal epithelial cells, revealing a targeted strategy to breach mucosal barriers (Woodward et al., 2013). In contrast, transmembrane mucins (e.g., MUC16) counteract this process by masking entry receptors and limiting infections (Woodward et al., 2013). Together, these findings highlight a dynamic host–virus interplay at the GLX, positioning it as a critical battleground that dictates infection versus protection. Notably, pathological remodeling of the GLX may act as a double-edged sword simultaneously weakening the physical barrier while redistributing key sulfated motifs. Dynamic remodeling of the GLX during inflammation, aging, and chronic disease can generate transient windows of vulnerability in which loss of structural integrity exposes underlying receptors and facilitate pathogen attachment and entry. However, GLX remodeling is not exclusively detrimental and can also function as an adaptive component of host defense. In the respiratory tract, membrane-bound and secreted mucins (MUCs) represent key glycan-rich interfaces for viral interaction, including Influenza A virus, by presenting abundant sialylated motifs that serve both as attachment substrates and decoy receptors (Linden et al., 2008). These interactions impose evolutionary constraints on Influenza A virus, driving continual adaptation of hemagglutinin binding specificity and neuraminidase activity to navigate mucin-rich barriers and evade entrapment within airway mucus (Weber et al., 2026). Conversely, mucin shedding and release of soluble glycan-rich fragments can enhance antiviral defense by sequestering virions and promoting mucociliary clearance, thereby limiting productive infection (McAuley et al., 2017). Collectively, GLX remodeling reflects a context-dependent and bidirectional biological process that can either promote or restrict infection depending on the pathogen, tissue environment, and specific glycan architecture. Defining these pathogen-specific mechanisms of GLX exploitation and defense will be critical for developing therapeutic strategies that preserve protective barrier functions while preventing microbial hijacking of host glycans.

### 2.3 Glycosaminoglycans: charged polymer chains

GAGs are far from passive coatings; they are long, linear, and highly polydisperse polysaccharides composed of repeating disaccharide units - typically an amino sugar and a uronic acid that undergo extensive post-translational modification (Varki et al., 2022). This maturation results in chains carrying one of the highest negative charge densities in biological systems. While the GLX contains a cocktail of GAGs, including HS, chondroitin sulfate (CS), dermatan sulfate (DS), and hyaluronan (HA), HS stands as the primary mediator of viral attachment. Extending hundreds of nanometers into the extracellular space, HS chains create a “sensory forest” of staggering structural diversity. Through the coordinated action of sulfotransferases and epimerases, the cell generates a chemical mosaic where specific sulfation codes (N-, 2-O, 6-O, and 3-O sulfation) serve as high-affinity docking motifs for viral glycoproteins, such as HSV-1’s gC and gB (Shukla and Spear, 2001; Esko and Lindahl, 2001). The influence of HS begins long before physical contact, acting as a sophisticated biophysical guidance system. The dense negative charge of the GAG layer generates a potent electrostatic potential that extends into the surrounding medium, creating an electrostatic funnel (Aricescu et al., 2002; Tiwari et al., 2012). This interaction is governed by the Debye length - the specific distance over which a charge’s effect persists in a saline biological environment ensuring the virus is captured by the GLX rather than drifting past. This long-range attraction facilitates electrostatic steering, exerting a torque that orients the viral particle, so its binding pocket faces the membrane as it approaches (Paiardi et al., 2021). From a soft-matter physics perspective, glycosaminoglycan (GAG) chains behave as highly hydrated flexible polymers that, when sparsely distributed on proteoglycan cores, adopt a mushroom-like configuration but transition into an extended polymer brush regime as grafting density increases on molecules such as Syndecans (Milner, 1991). This conformational behavior is consistent with established polymer brush theory and has been widely applied to describe the biophysical properties of the cell-surface GLX, including steric repulsion, hydration forces, and membrane–GLX coupling (Paszek et al., 2014). In this state, chains are forced to extend outward to minimize steric and electrostatic repulsion. Consequently, for a virion to reach the plasma membrane, it must physically penetrate this brush, incurring a significant entropic and osmotic energetic penalty as it displaces the water of hydration and compresses the negatively charged chains (Cui et al., 2023). The magnitude of this physical barrier, the steric exclusion limit is defined by GAG length, grafting density, and cross-linking with other GLX components like mucins (Li and Liu, 2025). Beyond mere penetration, this interaction triggers extensive cellular remodeling, specifically the induction of filopodia (Lehmann et al., 2005; Chang et al., 2022). These actin-rich protrusions act as highways, allowing the virus to navigate the GLX forest more efficiently. By exploiting these structures, viruses achieve higher tracking velocities and spread faster both internally and to neighboring cells (Lauster et al., 2023; Dong et al., 2021). Ultimately, viral entry is a negotiated process where the biochemical affinity for a receptor must overcome the mechanical resistance of the GLX forest to establish a robust infection.

### 2.4 Glycoproteins: functional receptors embedded in the glycocalyx

Glycoproteins constitute a second major class of GLX components and include many of the canonical receptors and co-receptors exploited by viruses for host cell entry (Lehmann et al., 2005). In contrast to PGs, these membrane proteins are typically smaller in size but are extensively modified with complex N-linked and O-linked glycans. These glycans substantially contribute to the negatively charged, and highly hydrated carbohydrate-protein-lipid fiber meshwork of the GLX, including thickness, hydration, and structural heterogeneity, thereby shaping a dynamic and highly organized cell surface interface (Jin et al., 2021). Importantly, viral receptors are not freely exposed to a flat membrane surface but are embedded within a dense and heterogeneous GLX matrix. Their accessibility to viral ligands is therefore tightly regulated by the surrounding glycan canopy, which can impose steric hindrance, influence receptor conformation, and modulate lateral mobility within the membrane plane (Mockl, 2020). This spatial embedding has significant implications for the kinetics and thermodynamics of virus–receptor interactions. Productive viral entry often requires precise nanoscale alignment between viral glycoproteins and host receptors, a process that is frequently impeded by the glycan shield - a dense array of host-derived carbohydrates that mask conserved protein epitopes and limits immediate accessibility for binding (Targosz-Korecka et al., 2021). To overcome these constraints, viruses have evolved strategies to exploit the dynamic nature of GLX. Transient conformational fluctuations, or “breathing” motions, within the GLX can intermittently expose receptor binding domains, thereby facilitating viral attachment (Casalino et al., 2022). In addition, some viruses actively remodel the glycan environment through enzymatic activity. For example, viral neuraminidases cleave terminal sialic acids, reducing steric hindrance and promoting efficient release of progeny virions from infected cells by preventing re-binding to sialylated glycans (McAuley et al., 2019). These adaptive mechanisms enhance the likelihood of productive receptor engagement in an otherwise restrictive environment. Beyond acting as a barrier, the GLX also plays an active role in organizing receptors into specialized functional microdomains that promote viral entry or cell to cell spread. This organization is frequently mediated by the formation of galectin lattices multivalent networks generated through the binding of galectins to β-galactoside-containing N-glycans on glycoproteins (Garner and Baum, 2008). These lattices function as molecular scaffolds that regulate receptor residence time at the cell surface, limit endocytic turnover, and facilitate the cooperative clustering of receptors. Such clustering is often essential for triggering downstream entry pathways, including clathrin-mediated endocytosis and macropinocytosis, both of which are commonly exploited by viruses making them unique antiviral targets (Schmidt et al., 2024; Lujan et al., 2022). The specific glycosylation state of receptors, including the extent of N-glycan branching, terminal sialylation, and fucosylation further modulates these interactions. Increased N-glycan branching enhances galectin affinity, thereby stabilizing receptor clustering and potentially promoting multivalent viral attachment (Wasik et al., 2016). Conversely, extensive sialylation generates localized negative charge density that can influence viral binding in a context-dependent manner: it may serve as a primary attachment determinant for sialic acid-binding viruses such as influenza or alternatively act as an electrostatic barrier that limits access for other pathogens (Wasik et al., 2016). Collectively, these findings highlight that receptor function within the GLX is not governed solely by protein–protein interactions but is instead a highly coordinated and context-dependent process dictated by the surrounding glycan environment. The GLX thus serves as both a physical barrier and a regulatory interface that fine-tunes viral attachment, receptor clustering, and entry efficiency (Mockl, 2020).

### 2.5 Mucins: extended glycoprotein barriers

Mucins represent a specialized class of high-molecular-weight glycoproteins that are central to the structural and protective functions of GLX. These molecules are distinguished by exceptionally long extracellular domains composed of tandemly repeated sequences that are densely modified with O-linked glycans (Honigfort et al., 2021). Canonical membrane-associated mucins, including MUC1, MUC4, and MUC16, can extend several hundred nanometers from the plasma membrane, frequently surpassing the height of surrounding globular glycoproteins and heparan sulfate (HS) chains. Their extracellular domains are enriched in proline, threonine, and serine (PTS) residues, which promote extensive O-glycosylation and enforce a highly extended, semi-rigid “bottlebrush” architecture (Holmen et al., 2004). This unique structural organization minimizes backbone flexibility while maximizing spatial occupancy, making mucins the principal determinants of GLX thickness and mechanical resilience (Kearns et al., 2024). From a biophysical perspective, mucins generate a substantial steric exclusion volume at the cell surface. Even at moderate expression levels, their extended conformation significantly increases the effective height and density of the GLX, thereby imposing a physical barrier that restricts the approach of large macromolecules, including viral particles (Linden et al., 2008; Kearns et al., 2024). This phenomenon, often referred to as the “mucin shield,” effectively gates access to the plasma membrane by limiting the ability of viruses to reach high-affinity entry receptors embedded deeper within the GLX (Honigfort et al., 2021). In fact, mucin biopolymers have shown a great promise being a broad-spectrum antiviral agent (Lieleg et al., 2012). As a result, viral entry becomes not only a function of receptor binding affinity but also of the ability to penetrate or bypass this polymeric barrier. In addition to their role as physical barriers, mucins exhibit an important and complementary function as molecular decoys. Their dense and diverse glycosylation patterns, which frequently include terminal sialic acids, fucosylated motifs, and other complex glycan structures, provide abundant low-affinity binding sites for viral attachment (Kearns et al., 2024). This high-density glycan presentation can sequester viral particles in non-productive interactions within the outer layers of the GLX, effectively reducing the probability of engagement with functional entry receptors located closer to the membrane. Thus, mucins contribute to host defense not only by steric hindrance but also by diverting viral binding toward non-permissive sites. The functional consequences of mucin expression become particularly pronounced in pathological conditions. In diseases with chronic inflammation and stress, mucin expression is often markedly upregulated, leading to the formation of an abnormally thick and densely packed GLX - sometimes referred to as a hypertrophic mucinome. This altered GLX architecture has significant implications in diagnosis and prognosis in certain cancer (Kufe, 2009). On one hand, the expanded mucin layer can sterically shield tumor-associated antigens from immune surveillance, impairing recognition by T-cell receptors and other immune effectors (Bhatia et al., 2019). On the other hand, it profoundly reshapes viral attachment dynamics by increasing the likelihood of viral sequestration within the outer glycosylated GLX while simultaneously reducing access to *bona fide* entry receptors (Chatterjee et al., 2023). In such contexts, the interaction between viruses and the host cell surface becomes a highly constrained and inefficient process. Although viral particles may exhibit increased initial binding due to the abundance of glycan ligands on mucins, they can remain kinetically trapped within the extended polymer brush, unable to traverse the dense GLX to initiate membrane fusion or endocytic uptake. This highlights a critical trade-off in mucin biology where the baseline mucin expression is essential for maintaining an effective protective barrier, excessive accumulation can create a biophysical environment that not only impedes immune function but also fundamentally alters the mechanisms of pathogen engagement.

Collectively, these observations underscore the importance of tightly regulated mucin expression in maintaining GLX homeostasis. Mucins function as both a defensive sieve and dynamic regulators of cell surface accessibility, with their quantitative and qualitative variations critically shaping viral entry, immune recognition, and overall cell surface biophysics. Building on this principle, synthetic mucin gels have emerged as biomimetic materials designed to target HIV and HSV-2 transmission (Kretschmer et al., 2022). By closely replicating the viscoelastic and barrier properties of native mucus, these gels can effectively trap and inhibit STD pathogens. Compared to simple mucin solutions, they exhibit superior lubricity and enhanced prophylactic efficacy, highlighting their promise as next-generation antiviral lubricants.

### 2.6 Glycolipids and minor components

Although quantitatively less abundant than proteoglycans and glycoproteins, glycolipids particularly sialic acid–containing gangliosides such as GM1 and GD1a play a disproportionately important role in organizing the structural and functional properties of the GLX, including membrane microdomain formation, receptor clustering, and pathogen attachment (Lopez and Schnaar, 2009; Simons and Gerl, 2010; Schnaar et al., 2014; Campanero-Rhodes et al., 2007; Suzuki, 2005). These amphipathic molecules consist of a ceramide lipid anchor embedded within the outer leaflet of the plasma membrane and a glycan headgroup that extends into the extracellular space (Krengel and Bousquet, 2014). Despite their relatively small size compared to extended glycoproteins or mucins, glycolipids contribute significantly to the nanoscale architecture of the cell surface and to the spatial patterning of viral entry factors (Drake et al., 2017; Garg et al., 2008). A defining feature of glycolipids is their preferential partitioning into cholesterol- and sphingolipid-enriched membrane microdomains known as lipid rafts (Hanafusa et al., 2020). These ordered domains exhibit reduced membrane fluidity and increase lateral packing, creating discrete platforms that compartmentalize specific proteins and signaling molecules (Sanders et al., 2021). Within the context of viral entry, lipid rafts serve as organizational hubs that concentrate receptors, co-receptors, and intracellular signaling machinery, thereby facilitating efficient virus–host interactions. The spatial segregation imposed by these domains can enhance receptor avidity through clustering and promote the coordinated activation of downstream pathways required for membrane fusion or endocytic uptake. For many viruses and toxins, glycolipids are not merely auxiliary components but function as essential receptors or co-receptors that mediate critical early steps in entry. Notably, Simian Virus 40 (SV40) and several bacterial toxins exploit GM1 gangliosides as primary binding partners, effectively bypassing canonical proteinaceous receptors (Gianni and Campadelli-Fiume, 2012). Binding GM1 induces local membrane curvature and promotes the formation of tightly curved invaginations, initiating caveolae- or raft-mediated endocytic pathways. This process exemplifies a “lipid-driven” entry mechanism in which the physicochemical properties of the membrane, rather than specific protein conformational changes, govern internalization.

The relatively small size and membrane-proximal localization of glycolipids position them at the base of the GLX, beneath the extended networks formed by mucins and glycoproteins. As a result, they are often shielded by the overlying polymer brush and become accessible to viral particles only after partial penetration of the GLX. In this context, glycolipids particularly sialylated gangliosides such as GM can function as membrane-proximal determinants that stabilize viral attachment and facilitate productive entry following initial low-affinity interactions with distal glycans or decoy structures. This sequential engagement reflects the multistep nature of viral entry, in which binding progresses from weak, reversible attachment to higher-affinity interactions that promote membrane organization, receptor clustering, and internalization. Several viruses exploit such staged glycan-dependent entry processes, although the specific receptors vary by virus. For example, influenza viruses can engage sialylated glycoconjugates including glycolipids, while rotaviruses utilize ganglioside interactions during cell entry, and coronaviruses and dengue virus rely primarily on glycan-binding attachment factors and co-receptors that precede engagement of their primary protein receptors (Vrijens et al., 2019; Negi et al., 2023; Martinez et al., 2013; Tantirimudalige et al., 2022). This hierarchical engagement strategy ensures that successful infection depends on both overcoming steric barriers and achieving precise spatial coordination at the cell surface. Furthermore, glycolipid-mediated interactions can influence membrane biophysics in ways that actively promote viral entry (Nguyen et al., 2022). Ganglioside clustering can induce local membrane curvature, alter lipid packing, and modulate the mechanical properties of the bilayer, thereby lowering the energetic barrier for membrane deformation and vesicle formation. These effects are particularly relevant for viruses that rely on endocytic pathways, as they facilitate the transition from surface attachment to internalization (Qian et al., 2009). Collectively, these observations highlight that glycolipids, while often categorized as “minor” components based on abundance, serve as critical regulators of GLX organization and viral entry. Their ability to orchestrate membrane microdomain formation, act as direct binding partners, and modulate membrane curvature underscores their central role in the multistep negotiation between viruses and the host cell surface (Lopez and Schnaar, 2009; Ewers et al., 2010). Notably, this functional importance has been exploited therapeutically: a novel soluble mimic of the glycolipid globotriaosyl ceramide has been shown to inhibit HIV infection by competitively engaging viral binding sites, effectively blocking interactions with membrane-proximal glycolipids (Lund et al., 2006).

### 2.7 Extending the dynamic cloud: the protein corona of the glycocalyx

The GLX is not a static polymeric barrier but a highly dynamic adsorption–desorption interface that continuously exchanges components with its extracellular environment. In addition to its covalently anchored glycans and membrane-associated constituents, the GLX is enveloped by a fluctuating layer of non-covalently associated biomolecules, predominantly plasma proteins such as albumin, orosomucoid, and fibronectin (Tarbell and Cancel, 2016). These proteins do not simply diffuse past the cell surface; rather, they transiently adsorb glycan structures, collectively forming what is increasingly described as a protein corona at the cell interface. This protein corona fundamentally reshapes the physicochemical identity of the GLX. By associating with negatively charged glycosaminoglycans (GAGs) (Kuo and Paszek, 2021), these proteins can dynamically modulate the effective surface charge, or zeta potential, of the cell. Such modulation has direct consequences for electrostatic interactions at the nanoscale (Kuo and Paszek, 2021). In particular, alterations in surface charge density influence the Debye length, the effective range over which electrostatic forces operate in a biological medium thereby tuning the long-range electrostatic landscape that governs the initial approach of viral particles toward the membrane (Tarbell and Cancel, 2016). In this sense, the protein corona acts as a tunable electrostatic filter, capable of either enhancing or attenuating the “electrostatic funnel” that guides viruses through the GLX.

Beyond passive modulation of surface charge, adsorbed proteins play an active and often underappreciated role as biophysical intermediaries in viral attachment. For several viruses, direct engagement with cell surface glycans or protein receptors is not the sole or even primary mechanism of entry. Instead, hepatitis viruses can exploit a “hitchhiking” strategy, wherein they bind to soluble host factors such as Apolipoprotein E (ApoE) or utilization of Growth Arrest-Specific Protein 6 (Gas6) by Zika virus and other flaviviruses, which have already partitioned into the GLX matrix (Sanders et al., 2021; Gianni and Campadelli-Fiume, 2012; Zhang et al., 2025). These host-derived ligands effectively serve as molecular bridges, linking the virus to the cell surface without requiring immediate penetration of the dense glycan network. This bridging mechanism provides a distinct biophysical advantage. In this context, the protein corona transforms the GLX from a purely defensive barrier into a conditional gateway one that can be co-opted to facilitate viral entry under specific molecular contexts (Sanders et al., 2021; Gianni and Campadelli-Fiume, 2012; Jiang et al., 2012). Importantly, the composition of the protein corona is not uniform but varies significantly across tissues and physiological states (Mayordomo et al., 2025), thereby imparting a layer of environmental specificity to viral interactions. In the vascular endothelium, particularly under high shear flow conditions, the adsorbed layer is enriched in albumin, which contributes to maintaining a strongly negative surface charge and a repulsive electrostatic environment that can limit nonspecific pathogen adhesion (Hierro-Oliva et al., 2021). In contrast, during inflammation such as in the pulmonary epithelium the composition of the corona shifts toward more adhesive and pro-coagulant proteins, including fibrinogen and other acute-phase reactants (Kangro et al., 2022). These proteins can increase surface stickiness and, paradoxically, create favorable conditions for viral attachment by stabilizing interactions that would otherwise be transient or energetically unfavorable (Huang et al., 2018; Kwaan and Lindholm, 2021; Landau et al., 2022; Favier et al., 2016). Thus, the protein corona extends the functional boundary of the GLX beyond its structural components, transforming it into a context-dependent and environmentally responsive interface. Rather than acting solely as a barrier, the GLX through its associated protein cloud emerges as a dynamic regulator of viral tropism, capable of either restricting or facilitating infection depending on the biochemical composition of its surrounding milieu.

## 3 Viral entry beyond the lock-and-key model: the HSV case

### 3.1 The mechanical invasion

Classical “lock-and-key” models of herpes simplex virus entry posit that viral glycoproteins engage specific cellular receptors with high affinity to trigger membrane fusion or endocytosis (Woodward et al., 2013; Wright et al., 2009; Imai et al., 2011; Clement et al., 2006). While conceptually useful, this reductionist framework overlooks the stochastic, multivalent, and biophysically constrained nature of virus–cell interactions at the GLX. Notably, HSV-1 interacts with GLX components in a dynamic and sometimes opposing manner: the lectin galectin-3 maintains epithelial barrier integrity by cross-linking transmembrane mucins (Abidine et al., 2022); however, HSV-1 can directly bind galectin-3 to facilitate viral attachment independently of canonical receptors such as nectin-1 (Morizono et al., 2011). In contrast, transmembrane mucins, particularly MUC16, can sterically hinder these interactions and reduce viral infectivity (Abidine et al., 2022). Beyond static binding, single-particle tracking reveals that HSV-1 diffusion at the cell surface is critically regulated by glycosaminoglycans (GAGs), with heparan sulfate promoting confined motion and enhanced entry, and chondroitin sulfate permitting freer diffusion but hindering stable attachment (Abidine et al., 2022). Viral mucin-like domains within glycoprotein C further facilitate surface exploration and accelerate penetration (Abidine et al., 2022; Long et al., 2024). Together, these observations support a model in which HSV-1 entry is a spatiotemporally orchestrated process, integrating lectin-mediated adhesion, mucin-dependent shielding, and GAG-regulated diffusion, highlighting the GLX as both a barrier and a dynamic regulator of viral attachment and internalization (Woodward et al., 2013; Wright et al., 2009; Imai et al., 2011; Clement et al., 2006; Abidine et al., 2022). These findings have direct implications for antiviral strategies targeting viruses that exploit proteoglycan core proteins such as Syndecans and glypicans. Rather than simply inhibiting heparan sulfate (HS) chains, modulating HS biosynthesis may be more effective: heparin and certain xylosides, despite *in vitro* antiviral activity, can paradoxically stimulate HS production, reinforcing the GLX (Cheudjeu, 2021). This supports the concept that viral attachment depends on glycosylation at HS initiation sites, with viral-induced processes heparanase overexpression, Syndecans shedding, and enhanced endocytosis disrupting GLX integrity and facilitating infection. Such deregulation also links to broader pathophysiological effects, including virus-associated GLX degradation and metabolic dysregulation.

In summary, virus invasion is a multistep, energy-dependent process that occurs within the crowded, heterogeneous, and mechanically dynamic GLX. Traditionally viewed as a passive, carbohydrate-rich scaffold, the GLX was thought to merely concentrate viral particles via low-affinity glycosaminoglycan (GAG) docking, facilitating receptor engagement (Domma et al., 2024; Ziarnik et al., 2025; Cui et al., 2023). Emerging evidence, however, reveals the GLX as a polymer brush - a dense, hydrated network generating steric, entropic, and electrostatic forces that actively regulate viral diffusion, orientation, and residence time at the cell surface (Ziarnik et al., 2025; Cui et al., 2023). For enveloped viruses such as HSV, entry requires overcoming these biophysical barriers to achieve precise alignment between viral glycoproteins and host receptors. Intact, the GLX forms a soft, elastic “molecular cushion” that buffers mechanical forces, restricts access to membrane receptors, and enforces selective permeability, permitting small solutes while hindering viral penetration (Boulant et al., 2015). Disruption of this architecture through inflammation, vascular injury, or metabolic disease reduces steric and electrostatic resistance, increases permeability, and creates a microenvironment permissive to viral entry and propagation (Richter and Sanderson, 2023). Together, these findings reframe viral entry as a mechanically constrained and environmentally regulated process.

### 3.2 The biophysical constraints of entry

**FIGURE 1:**
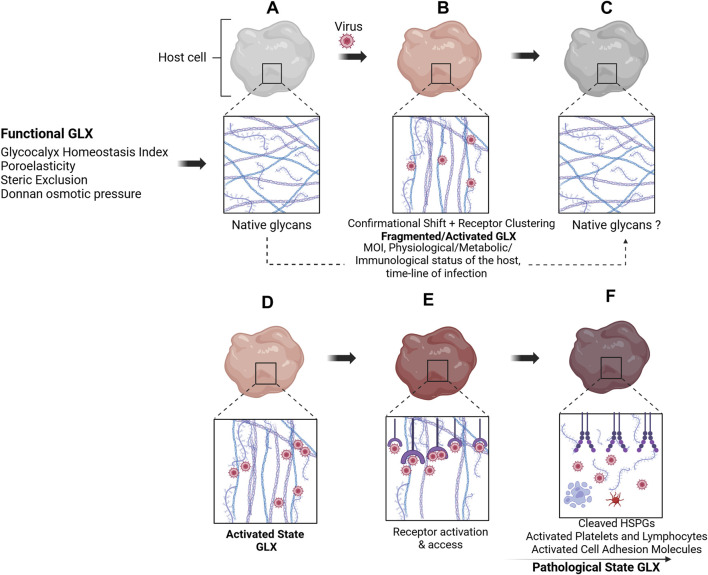
Viral-Driven Glycocalyx Remodeling and Host Recovery **(A)**. In healthy states, the GLX acts as a hydrated entropic barrier **(B)**. Viral encounter induces multivalent binding, triggering mechanical compression, glycan conformational shifts, and localized shedding that exposes entry receptors and amplifies pro-infectious signaling **(C)**. Host resilience depends on the balance between viral pressure (MOI/duration) and endogenous repair pathways that restore barrier function **(D–F)**. Viral engagement converts the GLX from a protective, anti-adhesive shield into a pathological, fragmented state characterized by HSPG cleavage, adhesion molecule exposure, and inflammatory cascade amplification, driving vascular and tissue dysfunction. Created in BioRender. Tiwari, V. (2026) https://app.biorender.com/citation/6a4bcb11c1a06d9ace971751.

From a physical perspective, the GLX imposes a fundamental distance constraint on viral entry that extends well beyond classical receptor–ligand considerations (Mockl, 2020; Cui et al., 2023). Viral adsorption, receptor accessibility, and fusion competency are all modulated by the need to overcome this barrier. Importantly, the GLX is not a rigid structure; it can deform, compress, and reorganize in response to external forces. During viral engagement, partial compression or displacement of the GLX may occur, altering local membrane mechanics and potentially triggering downstream signaling responses. Such perturbations can influence cytoskeletal organization, membrane tension, and receptor clustering, thereby coupling the physical act of viral entry with broader cellular responses (Chighizola et al., 2022). Although host cells possess intrinsic mechanisms to regenerate and maintain GLX integrity, the efficiency of this repair process is highly context dependent. Factors such as viral load, duration of exposure, and the host’s immune status play critical roles in determining whether GLX homeostasis can be restored. Under conditions of high multiplicity of infection (MOI), sustained viral challenge, or compromised immune function, the regenerative capacity of the GLX may be exceeded. In such cases, persistent thinning or fragmentation of GLX can occur, leading to increased membrane accessibility, dysregulated signaling, and heightened susceptibility to further viral invasion and tissue injury (Figure 1). Beyond its role as a physical barrier, the GLX functions as an active regulator of cellular mechanotransduction, signaling cascades, membrane curvature generation, and cell motility (Chighizola et al., 2022). These roles can be rigorously understood through the lens of polymer physics, which provides a quantitative framework for describing GLX behavior (Ziarnik et al., 2025). When modeled as a polymer brush, the GLX exhibits characteristic properties such as entropic elasticity, osmotic pressure, and resistance to compression. Together, these properties define a measurable energy barrier that incoming viral particles must overcome to reach the membrane surface (Ziarnik et al., 2025). A key insight from this framework is that membrane mechanics and surface glycosylation are intrinsically coupled. Variations in glycan length, density, and spatial organization do not merely correlate with membrane properties but actively determine them through steric and entropic forces characteristic of polymer brush systems. Increased glycan density or extension enhances steric repulsion, elevates the energetic cost of compression, and modulates membrane mechanical properties, including bending rigidity and curvature (de Gennes, 1980). These effects arise from the coupling between the GLX and the plasma membrane, where GLX architecture governs force transmission and membrane deformation (Squire et al., 2001). Consequently, the mechanical work required to bring a viral envelope into nanoscale proximity with the host membrane - a prerequisite for fusion, is directly governed by the architecture of the GLX. Thus, polymer-based models of the GLX move beyond descriptive analogies to provide predictive insight into viral entry. They enable a mechanistic understanding of how glycosylation patterns regulate membrane deformation, receptor accessibility, and fusion efficiency. In this view, viral entry emerges as a physically constrained process dictated not only by molecular recognition but also by the energetic and mechanical properties of the cell surface environment (Mockl, 2020; Ziarnik et al., 2025; Cui et al., 2023).

### 3.3 HSV as a case study in polymer physics

HSV entry exemplifies the integration of polymer physics, electrostatics, and conformational energy release in viral invasion (Jiang et al., 2012; Mayordomo et al., 2025). Prior to engaging high-affinity receptors such as Nectin-1, HVEM, or 3-O-sulfated HS, HSV must traverse the GLX (Spear, 2004; Shukla and Spear, 2001). The virus initiates attachment using glycoproteins gC and gB, which mediate a “soft landing” on the outer GLX through multivalent, low-affinity interactions with heparan sulfate. This initial docking increases virion residence time, allowing progressive penetration of the GLX despite extracellular flow. Electrostatic interactions further guide this process: the highly negatively charged sulfated GAGs create a local field that attracts positively charged domains on gC and gB (Valerio et al., 2025). This results in “viral surfing,” whereby HSV moves laterally along glycan chains (Oh et al., 2010), reducing the dimensionality of receptor search from three-dimensional space to two-dimensional surface diffusion, thereby increasing the likelihood of encountering an entry receptor. The transition from attachment to fusion is mechanically driven. Binding gD to its receptor triggers conformational changes that activate the gH/gL complex and the fusion protein gB (Eisenberg et al., 2012). The release of stored conformational energy performs mechanical work, displacing surrounding GLX polymers and enabling direct contact between the viral envelope and host lipid bilayer. In this way, HSV glycoproteins act as nanoscale actuators that actively remodel the local microenvironment to facilitate fusion. Host physiology further modulates this process. In inflamed tissues, the composition of the protein corona shifts toward pro-adhesive factors. HSV-1 induces tissue factor expression, triggering thrombin generation and promoting the conversion of fibrinogen to fibrin (Sutherland et al., 2007). In the case of SARS-CoV-2, fibrin has been shown to integrate into the GLX, forming a mechanically stable and adhesive scaffold that enhances viral attachment, shields the virus from immune recognition, and contributes to tissue damage (Kwaan and Lindholm, 2021). Therefore, HSV-1 may exploit a similar mechanism, whereby fibrin incorporation into the GLX helps protect the virus from immune detection and facilitates its interaction with host cells.

Overall, HSV entry illustrates how viruses exploit the physical, chemical, and mechanical properties of GLX. Viral success depends not only on receptor specificity but on overcoming steric, entropic, and electrostatic barriers while harnessing conformational energy to mechanically manipulate the host cell surface. This integrated perspective underscores the necessity of a unified biophysical framework for understanding viral entry (Eisenberg et al., 2012; Yamauchi and Helenius, 2013).

## 4 Pathological glycocalyx remodeling: shifting the biophysical threshold of infection

**FIGURE 2:**
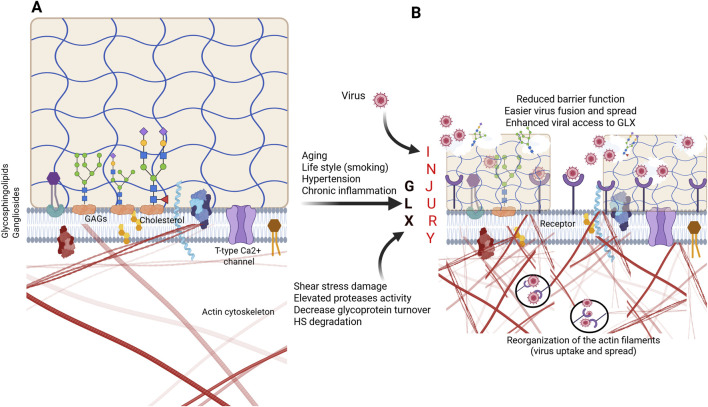
Mechanisms of Glycocalyx-Mediated Viral Susceptibility **(A)**. Healthy epithelial GLX sterically shields receptors and utilizes decoy glycan interactions to limit viral attachment **(B)**. Inflammatory, metabolic, and lifestyle stressors (e.g., smoking, hypertension) drive GLX thinning and altered glycosylation. This loss of structural integrity increases receptor accessibility, shifting the interface from a protective barrier to a permissive environment for viral internalization. Created in BioRender. Tiwari, V. (2026) https://BioRender.com/7ywtkih.

**FIGURE 3:**
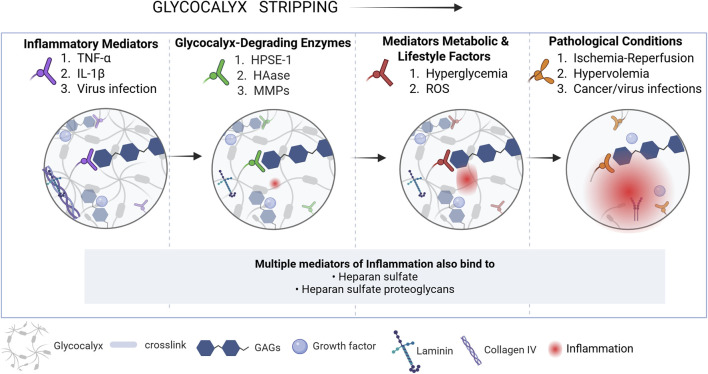
Enzymatic Dismantling of the Glycocalyx Barrier. Inflammatory and metabolic stressors (TNF-α, ROS, hyperglycemia) activate a synergistic network of degradative enzymes including heparanase, MMPs, and hyaluronidases to cleave core structural elements (HS, HA, Syndecan-1). This enzymatic stripping exposes endothelial adhesion molecules (ICAM-1, VCAM-1), promoting leukocyte recruitment and irreversible transition to a pro-inflammatory, dysfunctional surface. Created in BioRender. Tiwari, V. (2026) https://BioRender.com/tu27mdx.

The GLX is a dynamic, high-turnover biophysical interface whose structural integrity is exquisitely sensitive to physiological state (Ushiyama et al., 2016). Under pathological conditions, this highly organized polymeric landscape undergoes coordinated collapse, transforming the cell surface from a mechanically resistant barrier into a permissive, disordered interface that actively favors viral invasion (Figure 2). Importantly, this transition is not driven by receptor upregulation alone; rather, it reflects a system-wide reprogramming of steric, electrostatic, and mechanochemical constraints governing viral access. Inflammatory signaling serves as the primary upstream trigger of this transformation 24, (Masola et al., 2022). Pro-inflammatory cytokines induce a coordinated enzymatic remodeling program involving heparanases, hyaluronidases, and matrix metalloproteinases (Figure 3) (Masola et al., 2022; Martinez-Torres et al., 2004; Queisser et al., 2021; Schaaf et al., 2024), which collectively dismantle glycosaminoglycan chains and shed proteoglycan ectodomains. This process collapses the entropic and steric resistance of the GLX polymer brush, expanding a low-density peri-membrane zone that facilitates virion access to membrane-proximal receptors (Sharma et al., 2022). A central amplifier of this remodeling cascade is hyaluronan (HA), a non-sulfated glycosaminoglycan occupying both membrane-proximal and extracellular compartments of the GLX (Stern, 2004). Synthesized by hyaluronan synthase complexes (HAS1–3), HA is uniquely produced at the plasma membrane and can remain partially anchored at the cell surface while simultaneously extending into the extracellular space, thereby contributing to pericellular matrix organization, hydration, and viscoelastic buffering of the cell surface (Weigel et al., 1997). In this dual structural configuration, HA plays a key role in maintaining GLX spacing, macromolecular exclusion, and biomechanical resilience.

However, during inflammatory stress and oxidative injury, hyaluronidases and reactive oxygen species convert high-molecular-weight HA into low-molecular-weight fragments with distinct bioactive properties (Jiang et al., 2011). These fragments function not as inert degradation products but as potent damage-associated molecular patterns (DAMPs), engaging Toll-like receptor 4 (TLR4) and initiating MyD88-dependent signaling (Taylor et al., 2004). This rapidly amplifies NF-κB–driven transcriptional programs, reinforcing cytokine production and inducing additional matrix-degrading enzymes (Liu et al., 2017). The result is a self-propagating feed-forward loop in which enzymatic degradation and inflammatory signaling mutually reinforce GLX collapse, progressively lowering the biophysical threshold for viral entry.

Collectively, HA therefore functions not only as a structural element of the GLX but also as a dynamic mechanochemical regulator that links extracellular matrix integrity to inflammatory signaling intensity and susceptibility to pathogen access. Concomitantly, exposed membrane-proximal receptors such as Nectin-1 and HVEM become increasingly accessible, converting the cell surface into a high-affinity binding landscape for viral glycoproteins. In parallel, HA-derived DAMP signaling sustains local inflammation, ensuring persistent enzymatic activity and stabilizing a pathological state of GLX vulnerability (Lin et al., 2025). Superimposed on this inflammatory degradation axis is a systemic glycosylation switch characteristic of acute-phase responses. During the acute-phase response, inflammatory cytokines, particularly IL-6, induce the expression of glycosyltransferases, most notably ST6GAL1, resulting in a marked increase in α2,6-sialylation across circulating and cell-surface glycoproteins (Liu et al., 2018). This glycan remodeling reshapes electrostatic surface properties, modulates Siglec-mediated immune recognition, and dynamically alters receptor accessibility through changes in glycan architecture (Reitsma et al., 2007). Such inflammation-driven sialylation represents a rapid and highly adaptable layer of host glycan reprogramming that can influence both immune surveillance and pathogen interactions. In particular, increased α2,6-sialylation may alter the accessibility and organization of viral attachment factors and entry receptors, creating opportunities for exploitation by multiple enveloped viruses while simultaneously regulating innate and adaptive immune responses (Angata and Varki, 2023). Functionally, these changes establish a dynamic interface between inflammation, glycan biology, and viral pathogenesis, highlighting how systemic inflammatory states can reshape the molecular landscape encountered by invading pathogens.

In parallel, viruses actively exploit host glycosylation machinery during assembly and egress, generating envelope glycoproteins densely decorated with host-derived glycans that mimic endogenous glycan signatures. This molecular mimicry produces surfaces that function simultaneously as immune camouflage and lectin-engaging ligands. Such glycan-rich viral envelopes can also act as decoy-like structures that engage innate lectin pathways, including mannose-binding lectin (MBL) and DC-SIGN, thereby influencing viral recognition, clearance, and dissemination (Ip et al., 2009; Geijtenbeek et al., 2000). Importantly, viral glycan mimicry extends beyond passive shielding and reflects an active evolutionary strategy by which viruses exploit host glycosyltransferase activity to decorate viral glycoproteins with host-like glycans while simultaneously preserving or enhancing interactions with lectin-based attachment factors. This dual functionality positions viral glycans at the interface of immune evasion and receptor engagement rather than as purely structural modifications. For instance, heavily glycosylated viral proteins such as HIV gp120 acquire a dense host-derived glycan shield that both masks neutralizing epitopes and enhances attachment via lectin-mediated interactions with receptors such as DC-SIGN and mannose-binding lectin (Doores, 2015). Collectively, these glycan remodeling events highlight a bidirectional interface in which host inflammatory states reshape glycan topology while viruses exploit host glycosylation pathways to mimic immunological patterns, evade immune detection, and optimize entry efficiency.

Beyond acute inflammatory remodeling, chronic metabolic stress including hyperglycemia, obesity, and oxidative burden drives progressive GLX attrition (Ushiyama et al., 2016). This manifests as reduced glycan chain length, diminished polymer density, and loss of charge anisotropy, collectively compressing the spatial barrier between virions and membrane receptors. Concurrently, altered sulfation patterns weaken negative surface charge density, reducing electrostatic repulsion that normally governs long-range viral exclusion. Aging further compounds this process through reduced biosynthetic capacity and cumulative oxidative damage, resulting in mechanically fragile and compositionally simplified GLX architectures (Sun et al., 2023). These changes provide a biophysical explanation for increased viral susceptibility in elderly populations independent of classical immune decline. In this context, frailty highly prevalent in individuals aged 60–79 years - is increasingly associated with chronic viral burden, including cytomegalovirus (CMV) seropositivity (Araujo Carvalho et al., 2018).

Taken together, pathological GLX remodeling is best conceptualized not as a linear degradation process but as a nonlinear phase transition in cell surface biophysics, in which inflammatory signaling, enzymatic degradation, and glycan reprogramming converge to reshape the energetic landscape governing viral entry. This shifts the system from a high-barrier, low-probability infection regime to a low-barrier, high-probability state by simultaneously altering steric exclusion, electrostatic steering, and receptor accessibility. Within this framework, viral susceptibility emerges as an emergent property of GLX state variables polymer density, glycan composition, and charge distribution where even subtle perturbations can generate disproportionate increases in infection outcomes.

## 5 Viral strategies to exploit the glycocalyx landscape

While the GLX is a major biophysical barrier, many viruses exploit its molecular composition and mechanical properties for entry. Rather than acting solely as an obstacle, the GLX serves as a dynamic interface that facilitates viral capture, concentration, orientation, and mechanical priming (Domma et al., 2024; Ziarnik et al., 2025; Cui et al., 2023). Viral entry is therefore a coordinated multiscale process in which the GLX functions as a scaffold and navigational platform guiding membrane penetration. This process is further enhanced by viral multivalency, as seen in HSV], where repeated glycan-binding motifs strengthen interactions within the dense GLX, promoting stable attachment and deeper penetration without requiring enzymatic remodeling (Fukui et al., 2023).

Combined experimental and simulation data indicate that GLX architecture critically regulates viral entry. Atomic-force microscopy (AFM) studies using model GLX mimetics and cell-derived surfaces have demonstrated strong virus–GLX adhesion forces, supporting the role of the GLX in facilitating initial viral attachment (Ziarnik et al., 2025). However, the relationship between adhesion and productive entry is strongly dependent on GLX mechanics. In engineered or simplified systems, a flexible, brush-like or lightly cross-linked GLX permits local deformation around viral particles and can promote particle engagement and uptake, whereas increased cross-linking and mechanical rigidity reduce deformability and are associated with decreased viral penetration under both passive and externally applied forces (Ziarnik et al., 2025). Importantly, these mechanistic insights derive primarily from a combination of AFM-based measurements on model or reconstituted systems and are further supported by cellular studies in which GLX composition or stiffness is experimentally modulated. Computational and theoretical models of polymer-brush GLX behavior further support these observations but should be interpreted in the context of their simplifying assumptions regarding glycan heterogeneity and membrane coupling. This mechanical remodeling exposes membrane proteins, alters membrane rigidity, and promotes receptor nanoclustering, generating transient interfaces optimized for viral docking. For HSV-1 and other enveloped viruses, this proceeds as a sequential engagement cascade, where initial glycan binding serves as a priming step that aligns virions and synchronizes conformational changes in viral glycoproteins with receptor engagement, thereby reducing the total energetic cost of membrane fusion (Grove and Marsh, 2011). Viruses also exploit the intrinsic topographical heterogeneity of the GLX, preferentially targeting microdomains with lower steric density or favorable electrostatic profiles (Cui et al., 2023). Viruses exploit the intrinsic topographical heterogeneity of the GLX, preferentially engaging membrane microdomains characterized by reduced steric density, altered glycan composition, or locally favorable electrostatic environments. For example, influenza A virus preferentially binds clustered sialylated receptors within lipid raft–like domains, highlighting the role of membrane organization in viral docking and entry (Simons and Ikonen, 1997). Similarly, simian virus 40 (SV40) and other polyomaviruses exploit caveolae-associated membrane microdomains enriched in gangliosides such as GM1, which serve as critical entry platforms for internalization. Such spatially confined entry “hotspots” also arise from constitutive cellular processes, including clathrin-mediated endocytosis, which is utilized by viruses such as vesicular stomatitis virus (VSV), and macropinocytosis, which is exploited by Ebola virus, both of which depend on pre-organized membrane regions enriched in uptake machinery (Mercer et al., 2010; Doherty and McMahon, 2009; Mercer and Helenius, 2009; Bhattacharyya et al., 2010).

In parallel, mechanical forces such as shear stress can induce regional compression and thinning of the endothelial GLX, transiently exposing membrane-proximal receptors and increasing susceptibility to adhesion in a spatially heterogeneous manner (Weinbaum et al., 2007; Tarbell and Pahakis, 2006). In addition, cytoskeletal remodeling during entry further concentrates receptors and actin-associated signaling components into discrete membrane domains, as exemplified by herpes simplex virus (HSV-1 and HSV-2), which induces localized actin rearrangement and receptor clustering at entry sites to facilitate fusion and penetration (Campadelli-Fiume et al., 2012; Du et al., 2025; Sasivimolrattana et al., 2023). Collectively, these examples illustrate that viral entry is not a uniform surface event but instead occurs at dynamic, structurally and mechanically defined GLX niches that emerge from physiological membrane heterogeneity and are actively exploited by diverse viruses to optimize attachment and internalization. However, how these rapid and coordinated remodeling events unfold during the earliest stages of viral invasion, particularly across the transition from initial attachment to membrane penetration remains insufficiently understood and warrants further investigation.

Finally, viruses frequently exploit molecular mimicry by cloaking themselves in host-derived glycans, thereby adopting a “GLX-like” exterior. This strategy facilitates stealthy entry by masking pathogen-associated molecular patterns, attenuating lectin-mediated immune recognition, and prolonging viral residency at the cell surface (Miller et al., 2021). Collectively, these observations underscore that viral entry is not a discrete receptor-binding event, but rather an emergent property arising from the cooperative interplay between the virus and the host’s dynamic GLX, wherein chemical, mechanical, and topographical cues converge to govern the efficiency, orientation, and likelihood of productive infection. Notably, the behavior of viruses and their progeny within both functional and pathologically remodeled GLX environments remains poorly defined, representing a critical gap in current understanding. Given that most living cells are enveloped by a dense, carbohydrate-rich GLX composed of glycoproteins, glycolipids, and PG structures that play central roles in cell–cell communication, immune recognition, and pathogen–host interactions these glycoconjugates serve as critical determinants of viral attachment and entry. Accordingly, carbohydrate-based biomaterials (Cova et al., 2026), owing to their inherent biocompatibility and structural mimicry of native glycans, represent a powerful platform to modulate viral entry processes. Their ability to evade rapid clearance, recapitulate key glycan-mediated interactions, and competitively interfere with virus–host binding events positions them as promising tools for antiviral strategies, including decoy receptors, entry inhibitors, and targeted delivery systems.

## 6 Implications for antiviral design and synthetic biology

Antiviral design is increasingly shifting toward targeting the mechanical and spatial constraints that govern viral entry. Productive entry often requires compression of the GLX and receptor nanoclustering; accordingly, synthetic constructs can be engineered to stiffen the GLX, restrict receptor lateral mobility, or modulate membrane–cytoskeletal coupling to inhibit viral fusion even after initial binding (Ezzatpour et al., 2025). These mechanobiological strategies are particularly relevant in pathological contexts such as metabolic disease, chronic inflammation, or aging, where GLX thinning enhances susceptibility to infection. Additionally, interventions that promote GLX regeneration or inhibit shedding enzymes can restore barrier integrity, reduce viral access, and attenuate inflammatory signaling. Reframing viral entry as a biophysical, systems-level process shifts antiviral strategies away from discrete receptor–ligand blockade toward modulation of the broader cellular interface. Conventional antivirals targeting viral enzymes or specific receptors are often undermined by redundancy and rapid mutation, whereas GLX-focused approaches exploit conserved physical parameters such as steric hindrance, glycan density, and electrostatic interactions to reduce the probability of productive entry across diverse pathogens (Balzarini, 2007; Francois and Balzarini, 2012). This probabilistic framework is more accurately reflected *in vivo* infection dynamics. Synthetic biology provides a toolkit to implement this paradigm. Engineering PGs expressions can increase GLX thickness and elevate the energetic barrier to viral penetration, particularly in mucosal and epithelial tissues (Purcell and Godula, 2019; Chighizola et al., 2022; Huang et al., 2016). For example, in the case of SARS-CoV-2, an intact and thick GLX serves as a critical protective barrier that limits viral access to endothelial cells (ECs). By maintaining physical separation between circulating virions and membrane-associated receptors, the GLX reduces productive interactions between the viral spike protein and angiotensin-converting enzyme 2 (ACE2). Conversely, disruption or degradation of the GLX exposes underlying receptors, facilitating spike protein binding, viral entry, and subsequent endothelial injury (Lv et al., 2023). Furthermore, the hyperinflammatory response associated with severe COVID-19, often referred to as a cytokine storm, can directly damage the GLX through the induction of GLX-shedding enzymes, oxidative stress, and inflammatory mediators (Lv et al., 2023). Progressive loss of the GLX contributes to endothelial dysfunction, increased vascular permeability, leukocyte adhesion, and activation of procoagulant pathways, ultimately promoting the thrombotic complications frequently observed in severe disease (Lv et al., 2023). Collectively, these findings identify the GLX as both a target and regulator of COVID-19 pathogenesis and suggest that preservation or restoration of GLX integrity represents a promising therapeutic strategy to limit viral invasion, protect endothelial function, and mitigate inflammation-driven vascular dysfunction and coagulopathy during SARS-CoV-2 infection (Lv et al., 2023).

Complementary strategies include glycan decoys and synthetic polymer scaffolds that sequester virions through multivalent, low-affinity interactions, thereby limiting access to the plasma membrane (Elste et al., 2025; Ziarnik et al., 2025). In parallel, modulation of polymer brush elasticity and cytoskeletal coupling offers a route to disrupt the mechanical prerequisites of entry. HSV exemplifies these principles. HSV relies on initial HS interactions to concentrate at the cell surface; thus, altering HS density, spatial organization, or sulfation patterns can significantly impair infection efficiency. Engineered cell surfaces and polymer brush models are advancing this field by transforming viral entry from a descriptive phenomenon into a predictive and engineerable process. For example, synthetic mucin-mimetic polymer brushes have been used to systematically vary glycan density, chain length, and steric architecture, revealing how GLX crowding regulates access of pathogens and ligands to membrane-proximal receptors (Delaveris et al., 2020). In the case of influenza A virus, synthetic glycan arrays and engineered sialylated surfaces have been widely employed to define receptor specificity and predict host tropism based on glycan-binding preferences (Stevens et al., 2006). Likewise, glycoengineering approaches have demonstrated that SARS-CoV-2 spike protein utilizes cell-surface heparan sulfate as a cofactor for ACE2 engagement, illustrating how GLX architecture can directly influence viral attachment and receptor accessibility (Clausen et al., 2020). Collectively, GLX-targeted strategies not only offer potential to prevent viral entry but also provide insight into GLX dynamics, which may inform broader preventive approaches to understanding and managing infectious diseases. Finally, the discovery of glycoRNA extends the scope of glycovirology by demonstrating that glycosylation is not limited to proteins and lipids but also occurs on RNA, thereby redefining the molecular landscape of host–virus interactions and opening new avenues for antiviral intervention and immunomodulation (Kang et al., 2026).

## 7 The glycocalyx: a central biophysical and glyco-immune interface

**FIGURE 4:**
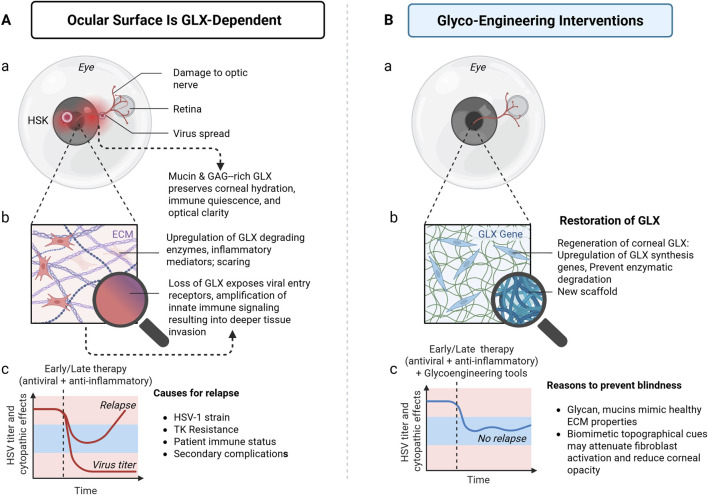
GLX Remodeling in Herpetic Eye Disease and Restoration **(A)**. HSV-1 infection disrupts the ocular GLX (panels a,b), facilitating viral spread and chronic inflammatory pathology (HSK) that current replication-centered therapies fail to resolve (panel c) **(B)**. Glycoengineering strategies including enzyme inhibition, biosynthetic upregulation, and biomimetic scaffolds (panels a–c) offer a multimodal approach to reconstitute barrier integrity and re-establish ocular homeostasis. Created in BioRender. Tiwari, V. (2026) https://BioRender.com/4ncr0yt.

**FIGURE 5:**
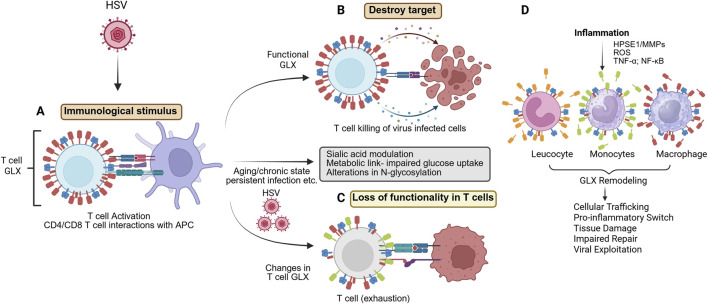
Glycocalyx Remodeling and Immune Dysfunction **(A–C)**. Chronic viral exposure and metabolic stress drive T cell exhaustion by altering surface sialylation and N-glycosylation. These changes disrupt immunological synapse stability and impair antiviral cytokine production **(D)**. In myeloid cells, GLX remodeling skews phenotypes toward hyperinflammatory signaling and enhanced trafficking, exacerbating tissue injury while diminishing reparative functions. Created in BioRender. Tiwari, V. (2026) https://BioRender.com/541art4.

HSV-1–associated keratitis is a leading cause of infectious blindness worldwide, characterized by corneal scarring, neovascularization, and chronic immunopathology (Kaye and Baker, 1996) (Figure 4). Current antivirals suppress viral replication but fail to restore epithelial barrier integrity, leaving the corneal surface structurally compromised and prone to recurrent infection. Notably, the role of different HSV-1 strains in degrading or remodeling the ocular GLX remains poorly understood, representing a critical gap in our understanding of virulence and pathogenesis. The corneal epithelial GLX, dominated by membrane-associated mucins (MUC1, MUC4, MUC16), extends hundreds of nanometers into the extracellular space, forming a dense polymer brush (Martinez-Carrasco and Sharma, 2025). This structure, enriched with poly-N-acetyllactosamine and terminal sialic acid residues, creates both steric and electrostatic barriers while organizing membrane proteins and supporting interactions with lectins such as galectin-3 (Argüeso et al., 2009). Overall, the GLX acts as a combined physical and biochemical barrier, limiting pathogen access while helping regulate epithelial immune responses. Disruption of this interface through viral enzymatic activity, host glycosidases, or inflammation-induced shedding transforms a repulsive, virus-resistant barrier into an adhesive substrate, promoting viral attachment, receptor engagement, and lateral spread (Argueso, 2020). HSV-1 has evolved sophisticated mechanisms to exploit this landscape. Glycoprotein C (gC) contains a mucin-like domain (MLD) that mimics host mucins, enabling multivalent, low-affinity interactions with host glycosaminoglycans (Altgarde et al., 2015). Loss of the MLD dramatically reduces viral lateral mobility, receptor access, and responsiveness to GAG-mimetic inhibitors (Long et al., 2024; Altgarde et al., 2015), highlighting its role as a molecular integrator of GLX mechanics. Through transient cycles of binding and release, HSV-1 effectively “surfs” the epithelial surface (Oh et al., 2010), exploiting the GLX as a two-dimensional transport network to locate entry receptors such as Nectin-1. This conceptualization of the GLX as an active regulator of both viral entry and immune signaling has reshaped therapeutic thinking, highlighting the need not only to inhibit viral replication but also to restore or re-engineer the GLX itself. Synthetic mucin-inspired polymers (MIPs), based on dendronized polysulfate scaffolds, recapitulate native mucin topology, charge, and steric exclusion. These constructs potently inhibit viral spread at nanomolar concentrations by reinstating excluded-volume effects, sequestering virions, and restoring decoy functions effectively “re-greening” the epithelial surface (Long et al., 2024). Beyond viral inhibition, such approaches rebuild the physical and functional integrity of the barrier. Precise reconstruction of glycan composition and organization represents the next Frontier. Fucosylated and sialylated epitopes dictate pathogen interactions, immune cell recruitment, epithelial repair, and stem cell niche dynamics. Advances in glycomics including mass spectrometry and lectin microarrays enable cell-type-specific glycosylation profiling, providing a blueprint for rational GLX engineering to restore homeostasis and immune balance. GLX remodeling also affects immune cells (Figure 5), where changes in GLX structure can increase cell adhesion, migration into tissue, and activation, thereby contributing to damaging inflammation in stromal keratitis. In addition, prolonged HSV exposure can drive T cell exhaustion and weaken antiviral immunity especially during herpetic eye disease (Coulon et al., 2020).

**FIGURE 6:**
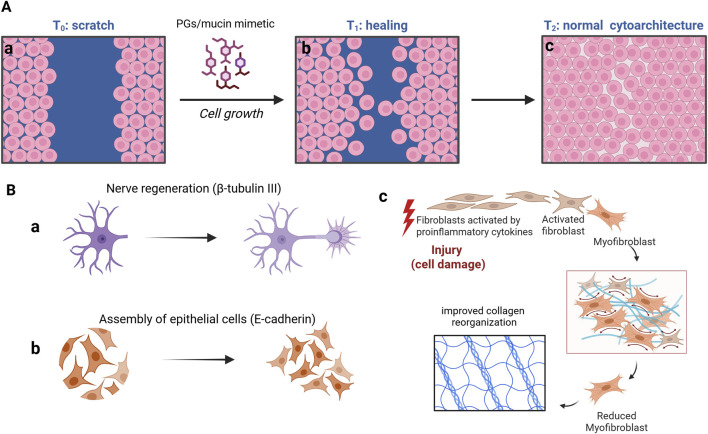
Glycocalyx Restoration Promotes Epithelial Repair **(A)**. HSV-1 disrupts GLX-mediated receptor shielding, impairing epithelial wound closure (cell migration and proliferation required to seal the injured area). Treatment with proteoglycan or mucin mimetics restores GLX integrity, accelerating barrier re-epithelialization **(B)**. GLX reconstitution enhances neurite outgrowth (a), suppresses myofibroblast differentiation (b), and promotes organized collagen deposition (c), collectively indicating a transition from viral injury to functional tissue repair. Created in BioRender. Tiwari, V. (2026) https://BioRender.com/kx2vcd2.

**FIGURE 7:**
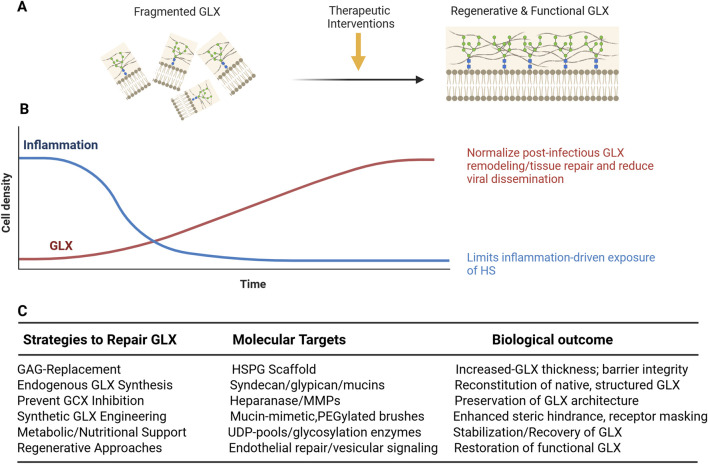
Therapeutic Strategies for Glycocalyx Reconstitution **(A–C)**. Targeted interventions aim to reverse inflammation-induced GLX fragmentation by suppressing pro-inflammatory mediators and inhibiting degradative enzymes. Multi-level restoration combines the supplementation of GAG mimetics with the stimulation of endogenous biosynthetic pathways to recover a dense, functional barrier and restore tissue homeostasis. Created in BioRender. Tiwari, V. (2026) https://BioRender.com/s38pt50.

Restoring the GLX offers a dual benefit: mucin- and heparan sulfate–rich surfaces can limit viral entry, mask damage signals, and reduce activation of inflammatory pathways such as NF-κB and MAPK. In this direction, wound-healing assays, along with advanced imaging and glycomic profiling, highlight how the GLX is dynamically disrupted during HSV-1 infection (Wilson, 2020; Burgalassi et al., 2011) (Figure 6), linking barrier structure, glycosylation, and immune responses. Overall, targeting the ocular GLX can both strengthen epithelial barrier integrity and better regulate local immune responses (Figure 7), offering a promising strategy for preventing and treating herpetic keratitis and positioning the GLX as an important focus in antiviral and regenerative medicine.

## 8 Outstanding questions and future directions

The spatial and compositional heterogeneity of GLX at functional length scales remains largely unresolved. While often modeled as a uniform polymer brush, emerging evidence reveals discrete microdomains with variable thickness, glycan composition, and charge density. How cytoskeletal dynamics, membrane curvature, and local glycan architecture define “entry-permissive hotspots” in HSV target cells is poorly understood, yet likely dictates why viral attachment and fusion occur at highly localized sites rather than uniformly across the surface. Equally critical is mapping the energetic landscape imposed by the GLX: the magnitude of steric, entropic, and electrostatic barriers, and how alterations in glycan length, branching, or sulfation modulate infection probability, are still undefined. Mechanistically, it remains unknown whether viruses remodel the GLX via direct mechanical forces or by recruiting host enzymes, or how rapidly these processes unfold relative to receptor engagement. Capturing these transient, nanoscale events will require high-resolution live-cell imaging, single-molecule force spectroscopy, and cutting-edge biophysical assays. Tissue specificity adds further complexity: epithelial, endothelial, and neuronal surfaces exhibit distinct GLX architectures, shaping viral tropism and entry strategies. Systematic mapping of this diversity promises to identify universal versus tissue-specific vulnerabilities across pathogens. Translationally, GLX integrity could serve as a predictive biomarker for susceptibility or disease severity, and noninvasive monitoring of GLX composition and mechanics could convert it into a clinically actionable target. Synthetic biology approaches offer unprecedented potential: programmable modulation of GLX thickness, stiffness, and glycan composition could reinforce barrier function while precisely tuning immune responses. Coupled with computational polymer physics models, such engineered GLX platforms would elevate viral entry from a descriptive observation to a predictive, engineerable process, establishing a transformative framework for host-directed antiviral strategies.

## 9 Concluding remarks: the glycocalyx as a universal biological nexus

Viral exploitation of the GLX reveals a fundamental principle of host–pathogen interactions: infection is dictated not merely by receptor engagement, but by the biophysical and biochemical architecture of the cell surface. Steric hindrance, electrostatic landscapes, and multivalent interactions collectively govern viral access, positioning the GLX as the primary gatekeeper of attachment and entry. As a viscoelastic and highly dynamic interface, the GLX functions as a barrier, sensor, and spatial organizer of receptor networks that ultimately determines infection outcomes. Yet, this same adaptability is subverted by viruses to facilitate adhesion, lateral mobility, and membrane fusion. Importantly, pathological remodeling driven by inflammation, aging, or metabolic stress disrupts GLX integrity, lowering the threshold for infection and enhancing cellular susceptibility.

Across diverse viral systems, the GLX emerges as a central determinant of infection strategy. In hepatitis C virus (HCV) infection, coordinated interactions between heparan sulfate proteoglycans particularly Syndecan-1 within the GLX and the tetraspanin CD81 drive efficient viral entry, while concurrent remodeling of GLX architecture indicates that dynamic reorganization of this layer is essential for productive infection. Similarly, in HSV-1, GLX-associated lectins such as galectin-3 are hijacked to enhance viral attachment, whereas transmembrane mucins such as MUC16 act as critical protective elements that mask entry mediators and restrict infection (Woodward et al., 2013). In flaviviruses, the viral NS1 protein induces tissue-specific disruption of endothelial GLX components, promoting vascular hyperpermeability and disease dissemination (Puerta-Guardo et al., 2019). Strikingly, respiratory syncytial virus (RSV) extends this paradigm further by incorporating the GLX into its own structural architecture (Lai et al., 2025). During viral assembly, RSV filaments become enveloped by and enriched with GLX components such as heparan sulfate and syndecan-4, effectively co-opting the host GLX as an integral part of the mature virion (Lai et al., 2025). Together, these findings redefine the GLX as a dynamic nexus governing viral attachment, entry, dissemination, and assembly. Despite significant progress, a critical gap remains in defining how the GLX preserves cellular homeostasis at both molecular and cellular scales. The fundamental principles by which complex system of GLX senses, integrates, and transduces extracellular cues into coordinated intracellular responses and how its disruption initiates and propagates disease remain largely unresolved. Bridging this gap will require a shift toward decoding the GLX as a functional system, not merely a structural entity. The ability to reconstruct and engineer a “healthy” GLX now represents a transformative Frontier (Masullo et al., 2025; Rana et al., 2026). Converging advances in synthetic glycobiology, organoid and organ-on-chip systems, high-resolution imaging, and computational modeling are poised to enable precise, systems-level control of this interface across infection, stress, and physiological conditions. In this context, the GLX can be re-envisioned as the molecular logic of the cell surface–a programmable and adaptive interface that governs cellular communication, environmental sensing, and response fidelity (Masullo et al., 2025). Decoding and harnessing this GLX-centric regulatory system at molecular and cellular levels will redefine our ability to intervene in disease. Such knowledge holds the potential to unlock next-generation strategies spanning infectious disease, cancer immunotherapy, organ transplantation, and regenerative medicine, while also enabling breakthroughs in targeted drug delivery and the precise modulation of cellular communication. Positioned at the nexus of structure, signaling, and function, the GLX is poised to emerge as a central paradigm in next-generation biomedical innovation.
